# Exploitation of blood non-Newtonian properties for ultrasonic measurement of hematocrit

**DOI:** 10.1038/s41598-021-89704-4

**Published:** 2021-05-13

**Authors:** B. Pialot, J. Gachelin, J. Provost, O. Couture

**Affiliations:** 1 Sorbonne Université, CNRS, INSERM, Laboratoire d’Imagerie Biomédicale, Paris, France; 2 ESPCI, CNRS, INSERM, PhysMedParis, Paris, France; 3 Aenitis Technologies, Paris, France; 4 Polytechnique Montréal, Montréal, Canada

**Keywords:** Biomedical engineering, Acoustics, Fluid dynamics

## Abstract

New processing techniques for manipulating blood and its components at a microfluidic scale are currently implemented. As for extracorporeal circulation, the in-line evaluation and monitoring of blood properties during these microfluidic techniques is a challenging task. Here, we show that the blood hematocrit can be measured non-invasively in a sub-millimeter medical tube using the non-Newtonian behavior of blood velocity profile. This hematocrit measurement is demonstrated on human blood with a simple Doppler ultrasound system. Results show a mean measurement error of 4.6 ± 1.3%Hct for hematocrit up to 52% and for 5 s-long ultrasonic signals. The simplicity and the measurement scale of the approach make it highly valuable for measuring hematocrit in new blood separation techniques. The approach may have an impact on in-vitro blood processing in general.

## Introduction

The field of in-vitro processing of flowing blood has been recently enlarged by the emergence of new separation techniques derived from microfluidics. These techniques are a major step toward a more effective and reliable production of blood products^1,2^. Also, extracorporeal circulation circuits such as hemodialysis^3^ or cardiopulmonary bypass^4^ are now widely available techniques to treat blood in-vitro. In these various cases, blood flows in sterile tubing, where it is difficult to monitor continuously its properties such as hematocrit without extracting a blood sample.

For cardiopulmonary bypass and hemodialysis, optical techniques are the most used modality for measuring hematocrit^5,6^. However, if optical sensors can be highly accurate, the required systems for their implementation are complex. Furthermore, optical approaches can be sensitive to red blood cells aggregation^7^. An alternative for measuring hematocrit is to exploit the dependence of blood electrical properties on red blood cells. Such electrical methods can be performed with simpler and more affordable systems than optic^8,9^ but they are highly sensitive to fluctuations of plasma properties and are thus not reproducible enough when applied to flowing blood^9,10^. For both optical and electrical systems, there is a lack of studies conducted in the context of microfluidic blood processing techniques. We can cite the systems of Zeidan et al*.*^11^ and Petersson et al*.*^12^ that are capable of measuring hematocrit in small channels. However, these two systems are respectively based on a flux cytometer and on a microscope and are thus hardly implementable in a compact set-up.

Ultrasound has the advantages of being low-cost and simple. Moreover, they preserve their coherence in complex media such as blood^13^. Contrary to optical and electrical approaches, their use for measuring hematocrit in-vitro has been less common so far. The use of blood acoustic attenuation has been yet demonstrated^14^ but the method has not been specifically designed for an in-vitro use. Also, the authors did not consider the dependence of acoustic attenuation on the shear rate^15^. We also demonstrated in a previous study that ultrasonic backscattered energy can be used to measure hematocrit in diluted blood from ultrasonic Doppler signals^16^.

It is well known that human blood is a pseudo-plastic non-Newtonian fluid whose apparent viscosity decreases with increasing shear rate^17^. It is furthermore generally admitted that non-Newtonian effects in human blood become preponderant under a shear rate of about 100 s^−1^^18,19^. These effects are influenced by blood composition and especially by plasma proteins but also by flow conduit structure, flow regime, pathologies and hematocrit^19,20^. From a theoretical point of view, the decrease of the apparent viscosity with the shear rate can be approximated by a power-law relationship. Such a model has been widely used even if it fails to correctly predict apparent viscosity when the shear rate tends to zero or infinity^20^. In particular, one of the most known power-law models is the one established by Walburn and Schneck^21^ on two hundred human blood samples using a cone-and plate viscometer. What makes the particularity of this power-law model is the incorporation of a hematocrit dependence that correlated significantly with experimental results^21,22^. The dependence of the power-law model upon blood constituents has been also investigated by Hussain et al*.*^23^. Their study confirmed that the parameters of the power-law model depend mainly on hematocrit but also highlighted the role of fibrinogen and cholesterol.

In this study, we present a simple ultrasonic system conceived for measuring the hematocrit of blood flowing in a sterile, sub-millimeter medical tube. The system is based on an easily implementable Continuous Wave (CW) Doppler ultrasound method that has been initially designed for an acoustophoretic blood separation device^16^. To perform the measure, the system exploits a decrease of the blood maximum Doppler frequency with hematocrit that can be derived from a power-law model analogous to the one of Walburn and Schneck^21^. The study is organized as follows. We first briefly present the general power-law model, its repercussion on the blood velocity profile and its link with the Doppler maximum frequency. Next, we describe the CW Doppler system and measurement procedures as well as the associated signal processing. Then, we experimentally characterize the decrease of the Doppler maximum frequency with hematocrit using a power-law model incorporating a hematocrit dependence. Finally, with human blood samples coming from different donors and for a fixed flow rate, we demonstrate how the system can be calibrated to measure unknown hematocrits.

## Materials and methods

### Ethics statement

All blood donors were healthy and adult volunteers who gave written and informed consent for this research study in accordance with the Declaration of Helsinki. Legal and ethical authorization for research use of collected blood was obtained through a national convention between the company Ænitis Technologies and the French Blood Institute (EFS) (convention number 15/EFS/028).

### Theory

We consider a laminar blood flow in a fixed tube of radius $R$ having a mean velocity ${v}_{mean}$ following a fixed imposed flow rate $Q=\pi {R}^{2}{v}_{mean}$ (in m^3^ s^−1^). We model the non-Newtonian behavior of blood by a power-law relationship, of which the expression is^20^

1 $${\mu }_{a}=k{\dot{\gamma }}^{n-1}$$

where ${\mu }_{a}$ is the blood apparent viscosity, $\dot{\gamma }$ is the shear rate and $k$ and $n$ are respectively the consistency index and the non-Newtonian index. From Eq. (1), it can be demonstrated that the one-dimensional non-Newtonian velocity profile for blood flowing in the tube is:

2 $$v\left(r\right)= \frac{3n+1}{n+1}{v}_{mean}\left[1-{\left(\frac{r}{R}\right)}^{1+\frac{1}{n}}\right]$$

where $v\left(r\right)$ is the blood velocity at the radial position $r$.

Now, we can simply write Eq. (2) for $r=0$ to establish a relationship linking the mean velocity ${v}_{mean}$, the maximum velocity ${v}_{max}$ and the non-Newtonian index $n$:

3 $${v}_{max}=\frac{3n+1}{n+1}{v}_{mean}$$

Next, we suppose that we insonify the blood flow with a CW Doppler ultrasonic probe. The probe is placed at an angle $\theta$ from the blood flow axis. The blood velocity $v(r)$ is then related to the Doppler frequency ${f}_{d}(r)$ of the ultrasonic signal by the Doppler equation^13^:

4 $${f}_{d}(r)=\frac{2{f}_{e}v(r)cos(\theta )}{c}$$

where ${f}_{e}$ is the ultrasonic frequency and $c$ is the speed of sound in blood.

With the combination of Eqs. (3) and (4), we finally have the same relationship between ${f}_{max}$ and ${f}_{dmean}$ than Eq. (3):

5 $${f}_{dmax}=\frac{3n+1}{n+1}{f}_{dmean}$$

### Experimental set-up

**Figure 1:**
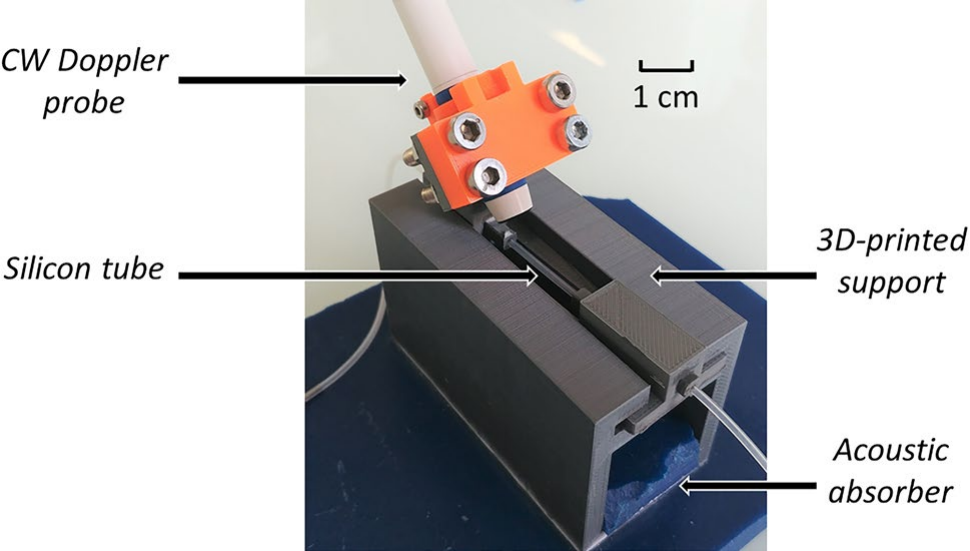
Picture of the probe and the tube mounted in the 3D-printed support.

An 8-MHz CW Doppler medical probe (diameter 1 cm) consisting of one emitter and one receiver was used for ultrasonic acquisitions. The blood was flowing into a 20-cm-long silicon medical tube (Freudenberg, Kaiserslautern, Germany, internal diameter: 760 µm) at a constant flow rate imposed by a syringe pump (KD Scientific Inc., Holliston, USA). The CW Doppler probe was positioned in a 3D-printed support at the tube half so that the blood velocity profile in the ultrasonic beam could be considered to be fully-developed. The Doppler angle between the CW Doppler probe and the tube axis was 40° and the entire cross-section of the tube was insonified. The entire set-up was immersed in water to ensure ultrasound propagation. A picture of the probe and the tube mounted in the 3D-printed support is shown in Fig. 1.

A function generator (Rigol, Beijing, China) was used to supply voltage to the probe. Backscattered ultrasonic signals were amplified (40 dB amplifier, Sofranel, Sartrouville, France) and digitized at 20 MHz on 8 bits with a USB 3.0 acquisition card (HS6, Tiepie engineering, Sneek, Netherlands). Post-processing was performed using Matlab (Mathworks, Natick, MA, USA). During the experiments, the function generator supplied a 10-V peak-to-peak voltage to the probe that led to a 10-kPa peak-to-peak ultrasonic wave.

For each blood sample, the reference hematocrit was measured with an automated medical analyzer (ABX Pentra 80, HORIBA, Kyoto, Japan). This medical analyzer combines focusing and optical counting of red blood cells to measure the hematocrit.

### Signal processing

All ultrasonic signals were acquired during 5 s and the following digital post-processing was performed for each signal. Signals were filtered using a Butterworth filter with a bandpass between 8 MHz − 5000 Hz and 8 MHz + 5000 Hz, underwent quadrature demodulation and were downsampled by a factor of 2000. To suppress unwanted spectral components below 80 Hz, a clutter filter was applied to each demodulated and downsampled signal. Signals were then segmented into 1-s segments for which corresponding Doppler power spectral densities (*PSD)* were estimated. The Doppler *PSD* of each signal was estimated by computing the median of the Doppler *PSD* of the 5 segments. Finally, a median filter of 40 Hz was applied to each Doppler *PSD* to reduce speckle noise.

### Influence of the flow rate

As non-Newtonian effects in the blood depend on shear rate and hence on flow rate for a given tube diameter, we first evaluated how the relationship between Doppler maximum frequency and hematocrit was for several flow rates.

Six blood samples at hematocrits between 5 and 53% were extracted from one blood bag diluted in its own plasma produced through centrifugation. Five acquisitions were performed for each sample and this for several flow rates ranging between 0.25 and 1.75 mL/min. The Doppler maximum frequency of each acquisition was determined using the model of Vilkomerson et al*.*^24^ following the procedure described in Pialot et al*.*^16^.

### Characterization of the relationship between the Doppler maximum frequency and hematocrit

We first supposed that the non-Newtonian behavior of blood flowing in our experimental set-up was similar to the one observed by Walburn and Schneck^21^. More precisely, we assumed that the blood apparent viscosity in our experimental set-up was ruled by a power-law with a non-Newtonian index $n$ that decreases with hematocrit such as:

6 $$n=1-CH$$

where $C$ is a coefficient to be determined experimentally. In the study of Walburn and Schneck^21^, the value of $C$ was estimated to 0.00499. Combining Eq. (6) with Eq. (5) we then had:

7 $${f}_{dmax}=\frac{4-3CH}{2-CH}{f}_{dmean}$$

Next, we evaluated the relevance of Eq. (7) for a fixed flow rate on 6 blood bags identified by B1, B2, B3, B4, B5, and B6. Each blood bag was diluted in its own plasma to obtain a total of 31 blood samples at hematocrits between 9.6 and 54.6%. Ten acquisitions were performed for each sample at a flow rate of 0.25 mL/min. For each acquisition, the Doppler maximum frequency was estimated using the Vilkomerson et al*.* model^24^. Finally, Eq. (7) was adjusted to the estimated Doppler maximum frequencies with ${f}_{dmean}$ and $C$ as fitting parameters.

A control experiment was conducted in order to verify that the blood mean velocity remained constant with increasing hematocrit. This experiment was performed using blood samples extracted from blood bag B5. For each sample, the total blood volume ejected at the output of the measuring tube during 4 min for a flow rate of 0.25 mL/min was precisely measured using a micropipette (0 to 1000 $\upmu$L capacity with a 10 $\upmu$L resolution). The flow rate corresponding to the measured blood volume was then computed and converted into blood mean velocity. The experiment was repeated three times for each blood sample.

### Definition of the $\tau$ parameter

As demonstrated by Vilkomerson et al*.*^24^, the Doppler *PSD* can be modeled by a function of a constant amplitude $A$ that breaks into a descending slope in the vicinity of the Doppler maximum frequency. More precisely, the Doppler maximum frequency is located at the half of this descending slope, where the amplitude of the *PSD* is $\frac{A}{2}$. Because of the value of 0.00499 for $C$ estimated by Walburn and Shneck^21^, we can certainly expect that the hematocrit-induced variations of this descending slope will be very thin. Hence, we chose to define another estimator that allows to estimate the hematocrit from the Doppler *PSD* without a precise consideration of the descending slope. This estimator can be derived as follow.

First, we can approximate the model of Vilkomerson et al*.*^24^ for the Doppler *PSD* by a sigmoid function $S$. One possible expression of such a function is:

8 $$S\left(f\right)=\frac{A}{2}\left(1+\mathrm{tanh}\left(\frac{{f}_{dmax}-f}{L}\right)\right)$$

where $A$ is the amplitude of the Doppler *PSD*, ${f}_{dmax}$ is the Doppler maximum frequency and $L$ is a parameter that rules the steepness of the descending slope of the Doppler *PSD*. This steepness corresponds to the spectral broadening effect and is a function of the acquisition time and of the dimensions of the ultrasonic probe.

The modeled *PSD* $S$ is correlated to hematocrit through ${f}_{dmax}$ but also through the amplitude $A$ which is a function of the blood backscattering coefficient^16^. One simple way to suppress this amplitude dependence of $S$ is to divide it by its mean value. If we denote by ${f}_{min}$ and ${f}_{max}$ the lower and upper bound of the frequency $f$, the mean value of $S$ is:

9 $$\mu \left({f}_{dmax}\right)=\frac{1}{{f}_{max}-{f}_{min}}\underset{{f}_{min}}{\overset{{f}_{max}}{\int }}S\left(f\right)df=\frac{A}{2}\left[1-\frac{L}{{f}_{max}-{f}_{min}}(ln\left({D}_{1}\right)-ln\left({D}_{2}\right))\right]$$

with:

$${D}_{1}=cosh\left(\frac{{f}_{dmax}-{f}_{max}}{L}\right), {D}_{2}=cosh\left(\frac{{f}_{dmax}-{f}_{min}}{L}\right)$$

In order to simplify the expression of $\mu$, we can use the approximations $ln\left({D}_{1}\right)\approx \frac{{f}_{max}-{f}_{dmax}}{L}-\mathrm{ln}(2)$ and $ln\left({D}_{2}\right)\approx \frac{{f}_{dmax}-{f}_{min}}{L}-\mathrm{ln}(2)$ that are reasonable if ${f}_{min}+L<{f}_{dmax}<{f}_{max}-L$. Equation (9) then becomes:

10 $$\mu \left({f}_{dmax}\right)=\frac{A}{2}\left[1+\frac{2{f}_{dmax}-{f}_{max}-{f}_{min}}{{f}_{max}-{f}_{min}}\right]$$

We can now obtain a *PSD* ${S}_{norm}$ independent of $A$ with the division of $S$ by $\mu$:

11 $${S}_{norm}\left(f\right)=B\left({f}_{dmax}\right)\left(1+\mathrm{tanh}\left(\frac{{f}_{dmax}-f}{L}\right)\right)$$

where $$B\left({f}_{dmax}\right)={\left[1+\frac{2{f}_{dmax}-{f}_{max}-{f}_{min}}{{f}_{max}-{f}_{min}}\right]}^{-1}$$.

The dependency of ${S}_{norm}$ toward hematocrit is only governed by ${f}_{dmax}$. Thus, any increase in the hematocrit, resulting in a decrease of ${f}_{dmax}$, will be followed by a proportional increase of the amplitude of ${S}_{norm}$. This increase of the amplitude can be simply quantified using the squared of the difference $\tau$ between ${S}_{norm}$ and its mean value of 1. The expression of $\tau$ is:

12 $$\tau ({f}_{dmax})=\underset{{f}_{min}}{\overset{{f}_{max}}{\int }}{\left(S\left(f\right)-1\right)}^{2}df$$

13 $$= \left({f}_{max}-{f}_{min}\right)\left(2{B\left({f}_{dmax}\right)}^{2}-2B\left({f}_{dmax}\right)+1\right)+2{LB\left({f}_{dmax}\right)}^{2}\left[tanh\left(\frac{{f}_{dmax}-{f}_{max}}{L}\right)-tanh\left(\frac{{f}_{dmax}-{f}_{min}}{L}\right)\right]+2B\left({f}_{dmax}\right)\left(B\left({f}_{dmax}\right)-1\right)\left(2{f}_{dmax}-{f}_{max}-{f}_{min}\right)$$

We will now consider the case where the spectral broadening of the Doppler *PSD* is negligible compared to its bandwidth. This assumption can be translated by $L\ll 1$. The expression for $\tau$ then becomes:

14 $$\tau ({f}_{dmax})=\left({f}_{max}-{f}_{min}\right)\left(2{B\left({f}_{dmax}\right)}^{2}-2B\left({f}_{dmax}\right)+1\right)+2B\left({f}_{dmax}\right)\left(B\left({f}_{dmax}\right)-1\right)\left(2{f}_{dmax}-{f}_{max}-{f}_{min}\right)$$

Finally, combining Eqs. (7) and (14), $\tau$ can be expressed as a function of the hematocrit $H$, the coefficient $C$ and the Doppler mean frequency ${f}_{dmean}$:

15 $$\tau (H,C,{f}_{dmean})=\left({f}_{max}-{f}_{min}\right)\left(2{B(H,C,{f}_{dmean})}^{2}-2B(H,C,{f}_{dmean})+1\right)+2B(H,C,{f}_{dmean})\left(B(H,C,{f}_{dmean})-1\right)\left(2\frac{4-3CH}{2-CH}{f}_{dmean}-{f}_{max}-{f}_{min}\right)$$

Using Eq. (15), values for $C$ and ${f}_{dmean}$ were estimated for the 6 blood bags such as in e) in order to compare the $\tau$ parameter approach with results given by the Vilkomerson et al*.* model^24^. The $\tau$ parameter of each blood sample was computed using trapezoidal integration following Eq. (12). Values for ${f}_{min}$ and ${f}_{max }$ were 80 Hz and 1000 Hz.

### Hematocrit measurement from Doppler PSD

Blood bags were divided into two groups: a “measuring” group built from blood bags B1, B4 and B5 and a “calibration” group built from blood bags B2, B3 and B6. The separation was performed randomly. First, calibration values for $C$ and ${f}_{dmean}$ were determined by fitting Eq. (15) to the $\tau$ parameters estimated for the calibration group. Then, calibration values for $C$ and ${f}_{dmean}$ where used to measure the hematocrit of blood samples from the measuring group using the following equation:

16 $$H=argmin{||\underset{{f}_{min}}{\overset{{f}_{max}}{\int }}{\left({S}_{m}\left(f\right)-1\right)}^{2}df-\tau (H,C,{f}_{dmean})||}_{2}$$

where ${S}_{m}$ is the measured Doppler *PSD* divided by its mean value. Values for ${f}_{min}$ and ${f}_{max }$ were again 80 Hz and 1000 Hz. This procedure was also performed using Doppler maximum frequencies and the Vilkomerson et al*.* model^24^ instead of $\tau$ for comparison.

## Results

**Figure 2:**
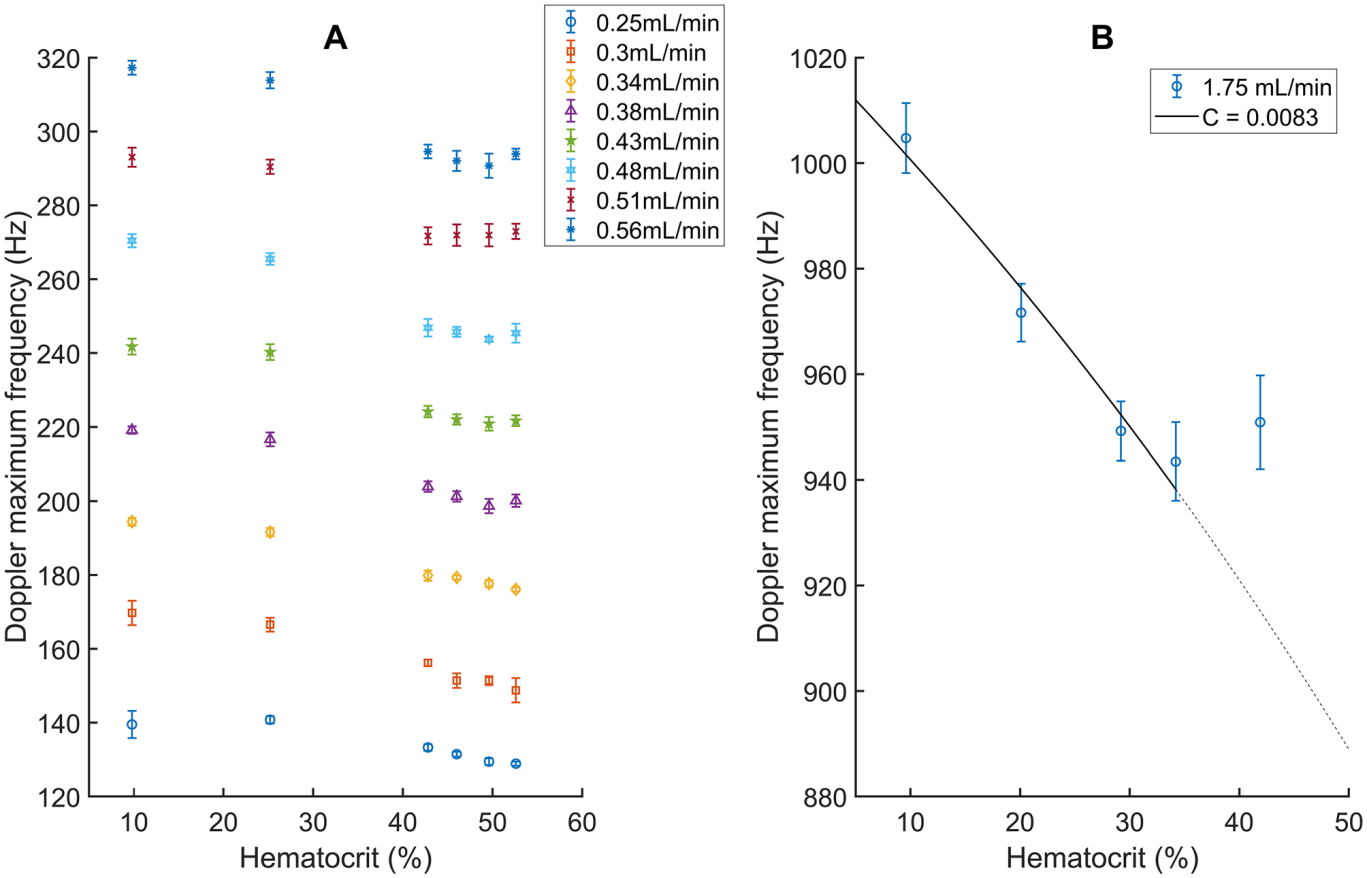
Experimental demonstration of the method flow rate dependency for the single tube radius used. The decrease of Doppler maximum frequencies saturates for an hematocrit superior to 50% if the flow rate is above or equal to 0.38 mL/min (**A**). The saturation effect has been observed for flow rates up to 1.75 mL/min (**B**). In (B) A fit of Eq. (7) has been performed but only for hematocrits where no saturation effect was observed.

Figure 2A shows the evolution of the relationship between the Doppler maximum frequency and the hematocrit as a function of flow rate. The Doppler maximum frequency decreases with increasing hematocrit but the decrease saturates at a hematocrit of 53%Hct when the flow rate crosses 0.38 mL/min. This saturation can be seen for all flow rates above 0.38 mL/min and seems to arise only when the hematocrit is above 40%Hct. This behavior is confirmed in Fig. 2B where we can see the same saturation effect for a flow rate of 1.75 mL/min.

**Figure 3:**
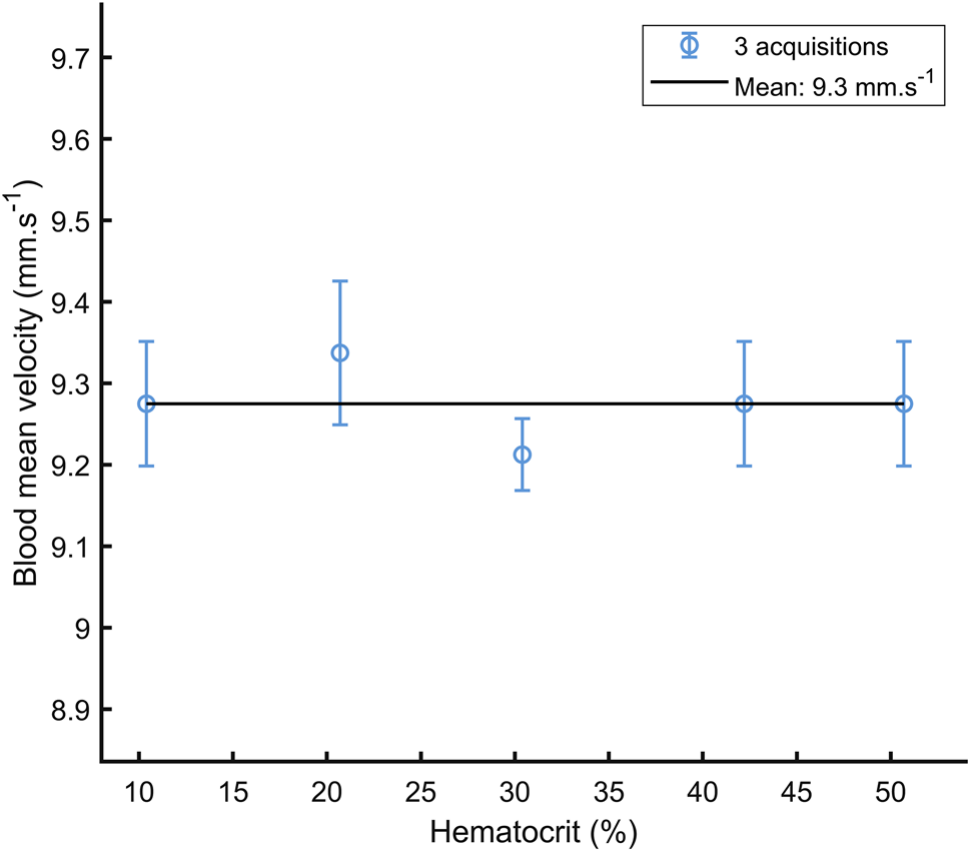
Measurement of mean blood velocity at a flow rate of 0.25 mL/min and for 5 hematocrit values. The measurement was performed from the blood volume ejected at the output of the experimental set-up during 4 min.

Figure 3 reports the blood mean velocity estimated with the micropipette. The blood mean velocity stays stable when the hematocrit increases. The mean velocity estimated on all hematocrits is 9.3 ± 0.1 mm s^−1^ (for an expected value of 9.4 mm s^−1^). The standard deviation of ± 0.1 mm s^−1^ converted in the Doppler frequency domain using Eq. (4) and a speed of sound of 1570 m s^−1^ is ± 0.4 Hz.

**Figure 4:**
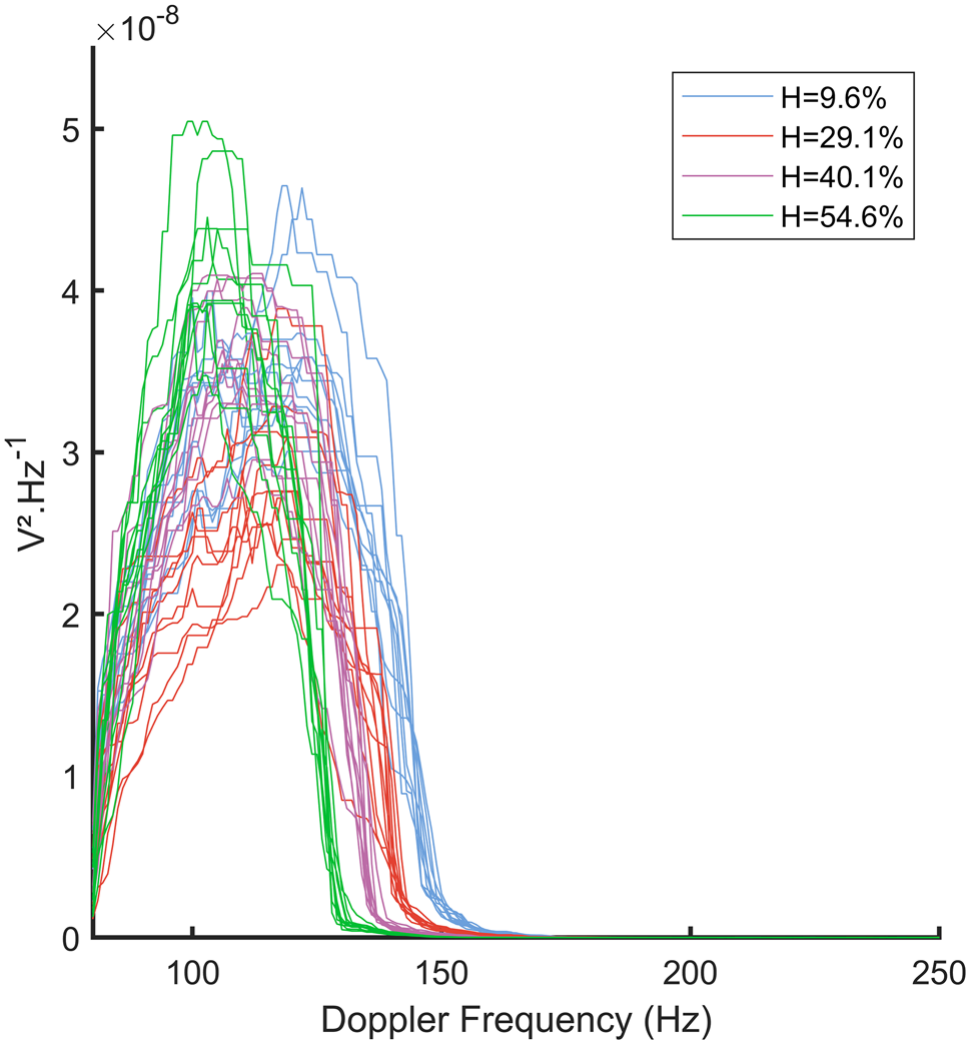
Variations of Doppler power spectral densities with hematocrit for one blood bag at a flow rate of 0.25 mL/min.

Measured Doppler *PSD* for blood bag B2 are reported in Fig. 4. The decrease of the Doppler *PSD* bandwidth with increasing hematocrit is clearly visible. We can also see that the Doppler *PSD* have a relatively high variance at the half of their descending slope (between 1.0 × 10^–8^ and 1.5 × 10^–8^ V^2^ Hz^−1^), which correspond to their Doppler maximum frequencies according to the model of Vilkomerson et al*.*^24^.

**Figure 5:**
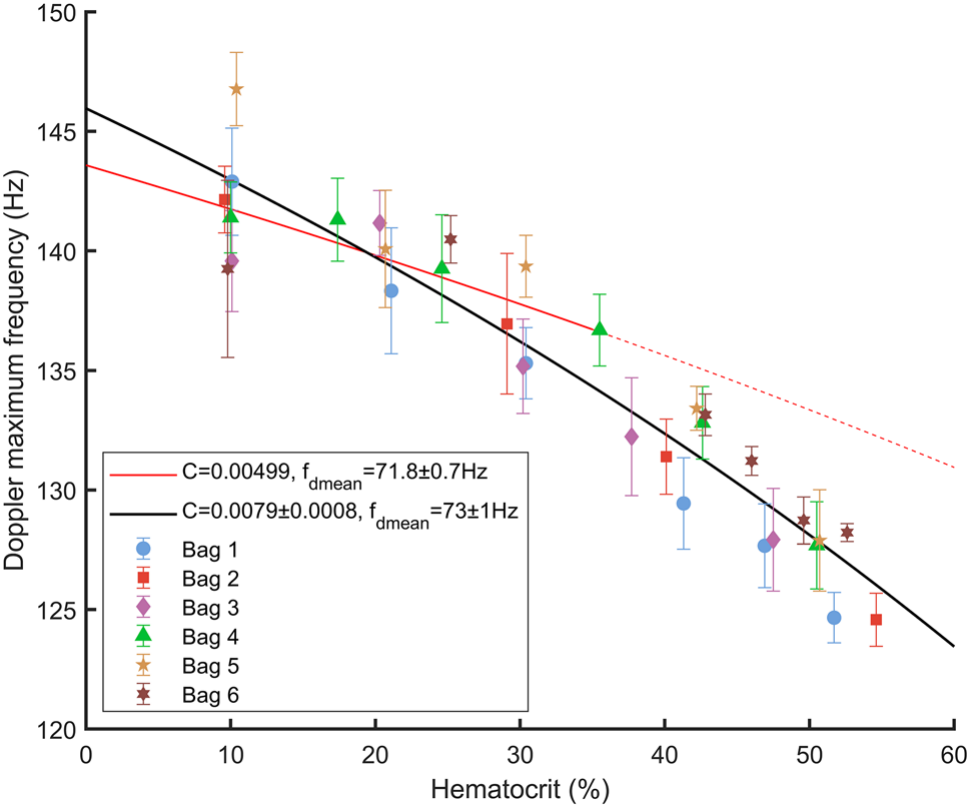
Measured Doppler maximum frequencies with a fit of Eq. (7) (black line) and with a fit of Eq. (7) restricted to hematocrits under 35% (red line). The fit on the whole hematocrit range has been performed with $\mathrm{C}$ and ${\mathrm{f}}_{\mathrm{dmean}}$ as fitting parameters and the restricted fit has been performed with ${\mathrm{f}}_{\mathrm{dmean}}$ as the only fitting parameter and with $\mathrm{C}$ fixed to the Walburn and Schneck value (1976).

As shown in Fig. 5, the measured Doppler maximum frequencies for the six blood bags follow a fit of Eq. (7) with $C$ and ${f}_{dmean}$ as fitting parameters. The value for $C$ is 0.0079 ± 0.0008 and the value for ${f}_{dmean}$ is 73 ± 1 Hz. The root mean square error of the fit is 2.3 Hz. Figure 5 also shows a fit of Eq. (7) but only for hematocrits under 35%Hct and with $C$ fixed to the value of 0.00499 estimated by Walburn and Schneck^21^. For this restrained fit the value of ${f}_{dmean}$ is 71.8 ± 0.7 Hz and the root mean square error is 2.7 Hz.

**Figure 6:**
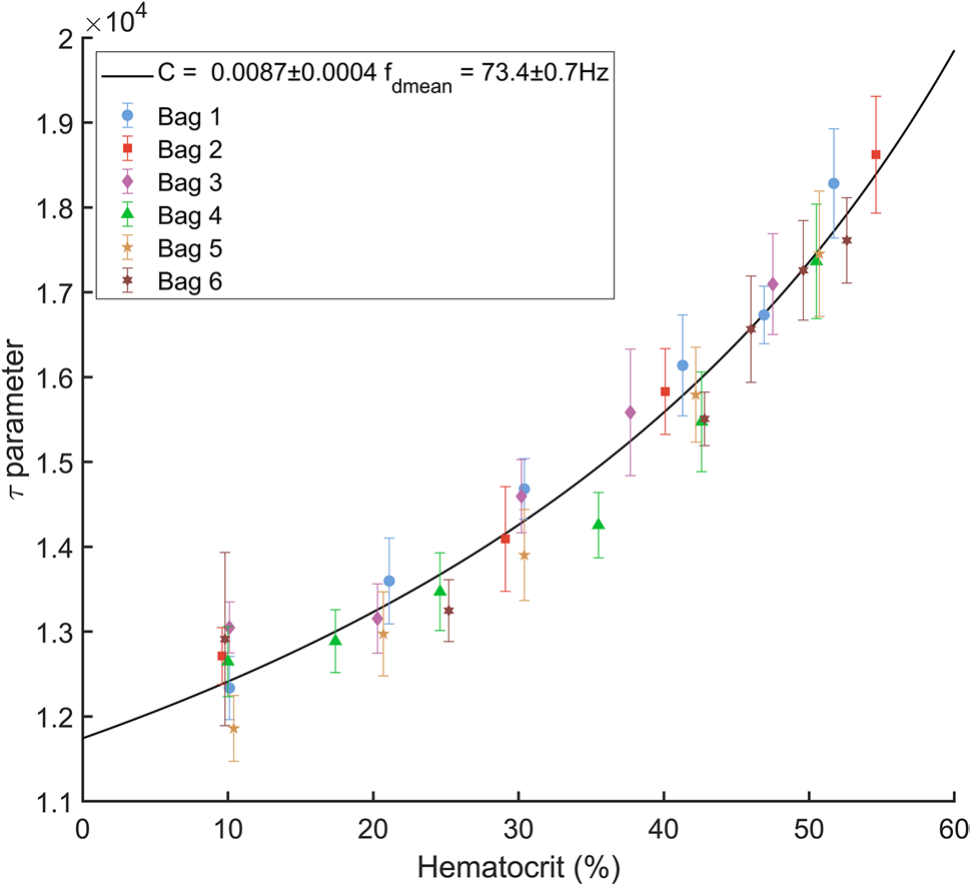
Measured $\uptau$ parameters with a fit of Eq. (15).

Figure 6 shows the measured $\tau$ parameters for the six blood bag with the fit of Eq. (15). The value for $C$ is 0.0087 ± 0.0004 and the value for ${f}_{dmean}$ is 73.4 ± 0.7 Hz. The root mean square error of the fit is 5.9.10^2^.

**Figure 7:**
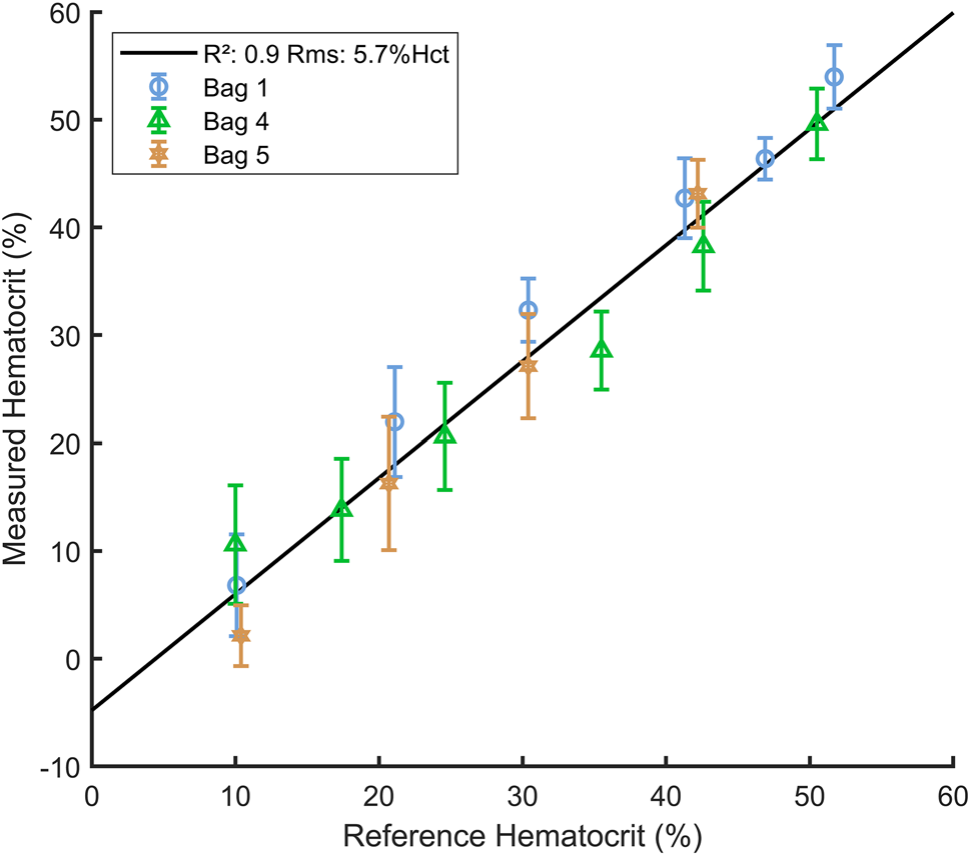
Hematocrit measurement for three different blood bags using the $\uptau$ parameter.

Finally, Fig. 7 reports the hematocrit measurement conducted on blood bags B1, B4 and B5 using Eq. (16) compared to values given by the ABX Pentra. The r^2^ coefficient of the linear regression between the two hematocrit estimations is 0.9 and the root mean square error is 5.7%Hct. The absolute error of the hematocrit measurement averaged on the 3 blood bags and using the ABX Pentra as a reference is 4.6 ± 1.3%Hct. Calibration values for $C$ and ${f}_{mean}$ determined on blood bags B2, B3 and B5 were respectively 0.0085 ± 0.0001 and 73.0 ± 0.3 Hz. In comparison, the absolute error of the hematocrit measurement using the Doppler maximum frequency estimated with the fit of the Vilkomerson et al*.* model^24^ is 6.7 ± 1.2%Hct.

## Discussion

We showed in this study how the non-Newtonian behavior of flowing blood can be exploited in order to measure its hematocrit. More precisely, our CW Doppler experimental set-up is able to detect slight decreases of the Doppler maximum frequency induced by the decrease of blood maximum velocity with increasing hematocrit. This decrease of the Doppler maximum frequency can be modeled with the assumption that blood behaves as a power-law fluid. Because of the high variance of the measured Doppler maximum frequency, we defined the parameter $\tau$ to more finely quantify the decrease of the Doppler signal bandwidth. With the use of this parameter, we showed on several blood bags coming from human donors how our experimental set-up can be calibrated for measuring unknown hematocrit with good accuracy.

The parameter $\tau$  is derived from several simplifications of the Doppler *PSD* shape. In particular, neglecting spectral broadening is realistic only for sufficiently long acquisition time and insonation length^24^. From our results, it is clear that the use of $\tau$ improves the accuracy of the hematocrit measurement. However, it also reduces the physical meaning of $C$ because of these simplifications but also because of the 80-Hz clutter filter which does not give access to the whole Doppler *PSD*. This loss of physical adequacy is highlighted by the difference between the value of $C$ estimated with $\tau$ and the one estimated with the Vilkomerson et al*.* model^24^. In the context of this study, we limited ourselves to the demonstration of the feasibility of a hematocrit measurement using the Doppler *PSD*. The development and the evaluation of an optimal estimator will be the subject of further experiments. Indeed, the accuracy at which the hematocrit-induced changes of the Doppler *PSD* can be detected are strongly dependent of acquisitions parameters. Ultimately, we can expect that the hematocrit could be measurable directly from the Doppler maximum frequency using the Vilkomerson model^24^ or any equivalent one. But, this improvement will necessite to raise our system signal-to-noise ratio in order to detect the descending slope of the Doppler *PSD* with less variance. Such an enhancement of the signal-to-noise ratio will be performed by using a higher sampling frequency, encoding ultrasonic signals on more bits and increasing the ultrasonic frequency.

For blood flowing at a constant mean velocity, the decrease of the Doppler maximum frequency is ruled by the $C$ coefficient. But, in the Walburn and Schneck model^21^ the value of $C$ was estimated to 0.00499 which is significantly lower than values estimated here. This result is very surprising considering that the coefficient $C$ should stay the same if the blood is in a non-Newtonian regime, independently of flowing conditions. To the authors knowledge, there is no study that examinated the correlation between hematocrit and blood velocity profile using a power-law. Hence, there is no available comparison in the literature. The fact that the Doppler maximum frequency decreases in a close way to the Walburn and Schneck model^21^ if hematocrit is restricted under 35%Hct suggests the occurance of another effect in addition to the increase of blood viscosity predicted by the power-law. The improvement of our system signal-to-noise ratio should help to clarify this eventuality. For the moment, we will simply retain that in our experimental set-up, the $C$ coefficient is stable and enables the measurement of hematocrit.

The observed saturation of the Doppler maximum frequency illustrates how the usability of our method will be affected by flowing conditions. Indeed, the decrease of blood maximum velocity will occur only if non-Newtonian effects are favorised. To highlight this imit, it is interesting to consider the blunting phenomenon.This phenomenom can be derived with Eq. (2), which shows that a decrease of the blood maximum velocity is accompanied by a blunting of the whole velocity profile. In this study, we did not observe the blunting because of the spatial averaging inherent to the CW Doppler technique. But, it is interesting to see what past studies on the blunting can reveal on the potential limits of our method. In particular, it has been observed that the blunting of the velocity profile in microtubes increases with decreasing tube diameter^25–28^. Also in microtubes, several studies have demonstrated that the blunting is inversely proportional to the shear rate^26–28^. Taking these observations as well as the saturation effect into account, we can expect that our method will suffer a trade-off between the tube radius and the flow rate for being generalizable. Interestingly, Yeleswarapu et al*.*^29^ have shown that a blunting of the blood velocity profile associated with a significant decrease of its maximum velocity can be observed for blood flowing at several cm s^−1^ in millimetric tubes.This tends to prove that in any case a valid compromise between tube radius and shear rate can be reached in higher scales than microfluidics. For future studies, it has to be noted that the use of particle image velocimetry to quantify more finely the blood velocity profile could be a valuable complement to our Doppler system. Another approach could be to use ultrasound localization microscopy to assess the velocity profile with microbubbles tracking.

The demonstrated measurement error is sufficient to show the feasibility of our method. However, our measurement sample is too restrained to fully conclude on the accuracy allowed by this experimental set-up. It has to be noted that our set-up has not been initially conceived to probe such small variations of Doppler signals bandwidth. Thus, the mean accuracy of our method could be certainly enhanced, notably by the improvement of the signal-to-noise ratio already mentioned. Also, Fig. 6 indicates that the method should be less accurate for hematocrit below 30%Hct if the $\tau$ parameter is used. This decrease of the accuracy arises mainly because of the slope of its curve with hematocrit wich is less steep below this limit. The same tendency is verified in Fig. 7 by the increase of the variance of hematocrit measurement below 30%Hct. But, in practice, that loss of accuracy is not a significant problem as a hematocrit of 30% is under the physiological range.

As demonstrated by Walburn and Schneck^21^ and by Hussain et al*.*^23^, the plasma composition plays a secondary role in non-Newtonian effects compared to hematocrit. This was confirmed by our experiments where the consideration of hematocrit only was sufficient to model the decrease of the Doppler maximum frequency. Thus, our method may have a fundamental advantage over electrical methods, that are sensitive to changes in plasma composition^9,10^. To compete with the most accurate optical systems, the accuracy of our experimental set-up will have to be enhanced. However, our approach has the advantage over optical methods to be usable with extremely simple and affordable material. Also, it has to be noted that the new blood processing techniques derived from microfluidics seem to be the most evident application of our method. Indeed, the small tubing and low flow rates used in these techniques will promote non-Newtonian effects and in particular the blunting of the velocity profile. The vast majority of microfluidic blood processing techniques aim at processing sterile blood samples where red blood cells are thus suspended in plasma. But, in some cases, the red blood cells might be altered or suspended in another diluent that could significantly affect blood behavior. This is for example the case in some extracorporeal blood treatments where a hemodilution is performed. In theses cases, our method will have to be re-evaluated. Also for extracorporeal circulation, a fundamental challenge to apply our method will be the very high flow rates and the tube diameters of several millimeters used in these techniques. Indeed, one should not forget that our method depends completely on the use of flowing conditions which are favorable to a non-Newtonian regime.

## Conclusion

We have presented a new method to estimate blood hematocrit in medical tubes. This method exploits the non-Newtonian behavior of blood with a simple ultrasonic CW Doppler system. The approach holds promises for in-vitro blood processing as it can be applied with a very simple ultrasonic apparatus. Many investigations are still needed before practical applications because of the complex physics of non-Newtonian effects. From a more general point of view, our study demonstrates that quantitative parameters can be extracted from the non-Newtonian behavior of flowing blood. This paves the way for more developed methods and models that may be used to characterize blood from this new perspective.

## Data Availability

The data that support the findings of this study are available from Aenitis Technologies but restrictions apply to the availability of these data, which were used under license for the current study, and so are not publicly available. Data are however available from the authors upon reasonable request and with permission of Aenitis Technologies.
